# Combination of acid β-glucosidase mutation and Saposin C deficiency in mice reveals *Gba1* mutation dependent and tissue-specific disease phenotype

**DOI:** 10.1038/s41598-019-41914-7

**Published:** 2019-04-03

**Authors:** Benjamin Liou, Wujuan Zhang, Venette Fannin, Brian Quinn, Huimin Ran, Kui Xu, Kenneth D. R. Setchell, David Witte, Gregory A. Grabowski, Ying Sun

**Affiliations:** 1 Division of Human Genetics, Cincinnati Children’s Hospital Medical Center, Cincinnati, OH USA; 2 Department of Pathology and Laboratory Medicine, Cincinnati Children’s Hospital Medical Center, Cincinnati, OH USA; 3 Department of Pediatrics, University of Cincinnati College of Medicine, Cincinnati, OH USA

## Abstract

Gaucher disease is caused by mutations in *GBA1* encoding acid β-glucosidase (GCase). Saposin C enhances GCase activity and protects GCase from intracellular proteolysis. Structure simulations indicated that the mutant GCases, N370S (0 S), V394L (4L) and D409V(9V)/H(9H), had altered function. To investigate the *in vivo* function of *Gba1* mutants, mouse models were generated by backcrossing the above homozygous mutant GCase mice into Saposin C deficient (C*) mice. Without saposin C, the mutant GCase activities in the resultant mouse tissues were reduced by ~50% compared with those in the presence of Saposin C. In contrast to 9H and 4L mice that have normal histology and life span, the 9H;C* and 4L;C* mice had shorter life spans. 9H;C* mice developed significant visceral glucosylceramide (GC) and glucosylsphingosine (GS) accumulation (GC»GS) and storage macrophages, but lesser GC in the brain, compared to 4L;C* mice that presents with a severe neuronopathic phenotype and accumulated GC and GS primarily in the brain. Unlike 9V mice that developed normally for over a year, 9V;C* pups had a lethal skin defect as did 0S;C* mice resembled that of 0S mice. These variant Gaucher disease mouse models presented a mutation specific phenotype and underscored the *in vivo* role of Saposin C in the modulation of Gaucher disease.

## Introduction

Acid β-glucosidase (GCase), encoded by *GBA1*, is the lysosomal hydrolase that hydrolyzes glucosylceramide (GC) and glucosylsphingosine (GS) to ceramide and sphingosine, respectively^1^. Disruptive *GBA1* mutations are causal to Gaucher disease by leading to insufficient GCase function and resultant GC and GS accumulation^1^. Three types of Gaucher disease are clinically defined, based on age at disease onset and organ involvement. Type 1 is the non-neuronopathic variant with highly variable visceral disease^2^. Type 1 patients also have an increased life-time risk of developing Parkinson disease variants indicating that mutations in *GBA1* are genetic risk factors^3^. Types 2 and 3 have early onset of primary, but variable, central nervous system (CNS) degeneration and are distinguished phenotypically by the rapidity of CNS disease progression in type 2 during the first year of life^4^. Several hundred *GBA1* mutations have been identified in affected patients^2,5^. Genotype/phenotype correlations show that the presence of homozygosity or heteroallelism of N370S predicts absence of early onset progressive CNS disease, and L444P homozygosity predisposes to variable primary CNS disease^6^. However, the relationships of other *GBA1* mutations and disease phenotypes are poorly understood.

In humans, the presence of the N370S allele in Gaucher disease patients is associated with type 1 and highly variable visceral involvement^2,7,8^. The D409H alleles have significant frequency and homozygotes (designated here as 9H) manifest early onset of variable visceral and the CNS involvement^9^. 9H patients also uniquely manifest calcific aortic root and valvular disease^10^. The V394L allele in humans has been reported only in the heteroallele state and is associated with type 1 or types 2 and 3 depending on the heteroallele^11^. In mice, *Gba1* mutations have been created to mimic those found in human patients^12^. In comparison to humans, N370S homozygosity (designated here as 0S) in mice leads to death within 24 hours due to a defect in the skin permeability barrier^12^. This is likely due to the lesser hydrolytic efficiency of the N370S enzyme toward the longer chain fatty acid acyl moieties on GC in murine skin vs. human^13–15^. Mice homozygous for V394L (designated here as 4L) and 9H have defective GCase activity and survive up to 2 years with relatively mild visceral abnormalities^12,16^. The mild phenotype in such murine models limits our understanding of *in vivo* effect of the *GBA1*/*Gba1* mutations.

Human GCase protein structures have been solved by X-ray crystallography and only one mutant GCase structure, N370S, has been characterized by X-ray and biochemical analyses^13,17,18^. The approach of studying protein - structure-function relationship has relied on structural modeling and dynamic simulation based on the crystal structure information^19,20^. A computation tool (Swiss-Pdb Viewer) can be applied to simulate *GBA1* mutations and their dynamic alterations, side chain interactions and force field energy changes at the atomic resolution for better understanding mutant GCases protein function.

Saposin C is a lysosomal protein that functions in maximizing enzymatic activity of GCase and in protecting GCase against intracellular proteolysis^21–23^. Mutations in the Saposin C region of the prosaposin gene (PSAP) produce variant forms of Gaucher disease^24,25^. Specific Saposin C deficient mice (C−/−, designated here as C*) were generated by a knock-in point mutation within the Saposin C domain of the Prosaposin locus (*Psap*), preserving Saposin A, B, and D, but leading to undetectable Saposin C protein and reductions of GCase activity and protein, and a slowly progressive CNS phenotype developing after 8–12 months^26^. Mice with Saposin C deficiency (C*) and homozygosity for 4L (combined model designated 4L;C*) have greater reductions in 4L GCase levels and develop a severe CNS disease phenotype compared to C* mice^26,27^.

To study the pathogenic effect of *Gba1* mutations and gain insights into the *in vivo* effects of GCase and Saposin C, additional *Gba1* mutant mouse models (0S, 9H, and 9V) combined with C* (designated as “*Gba1* mutation”;C*,. e.g, 0S;C*) were created and analyzed for biochemical, histopathologic, and phenotypic abnormalities. Compared to the models of 4L or 9H homozygotes combined with a hypomorphic prosaposin transgene that expresses subnormal levels of mouse prosaposin and four saposins^22^, these models allow the study of Saposin C’s specific effects with GCase mutants. These studies reveal mutation-dependent and tissues-specific phenotypes in *Gba1* mutant mice that are deficient in Saposin C and also highlight the critical role of Saposin C, or its potential variants, in GCase function.

## Results

### Simulation analysis of structural effects of GCase mutations

To understand differential effects of mutations on GCase conformation and function, human crystal structures^13,17,18^ were used to simulate the effects of D409H, D409V, V394L and N370S on GCase by analyzing side chain interactions and force field energy changes. In force field energy computing, negative energy values mean favorable energy environment, whereas positive values indicate unfavorable energy environment for a given amino acid (Supplementary Table 1).

**Figure 1:**
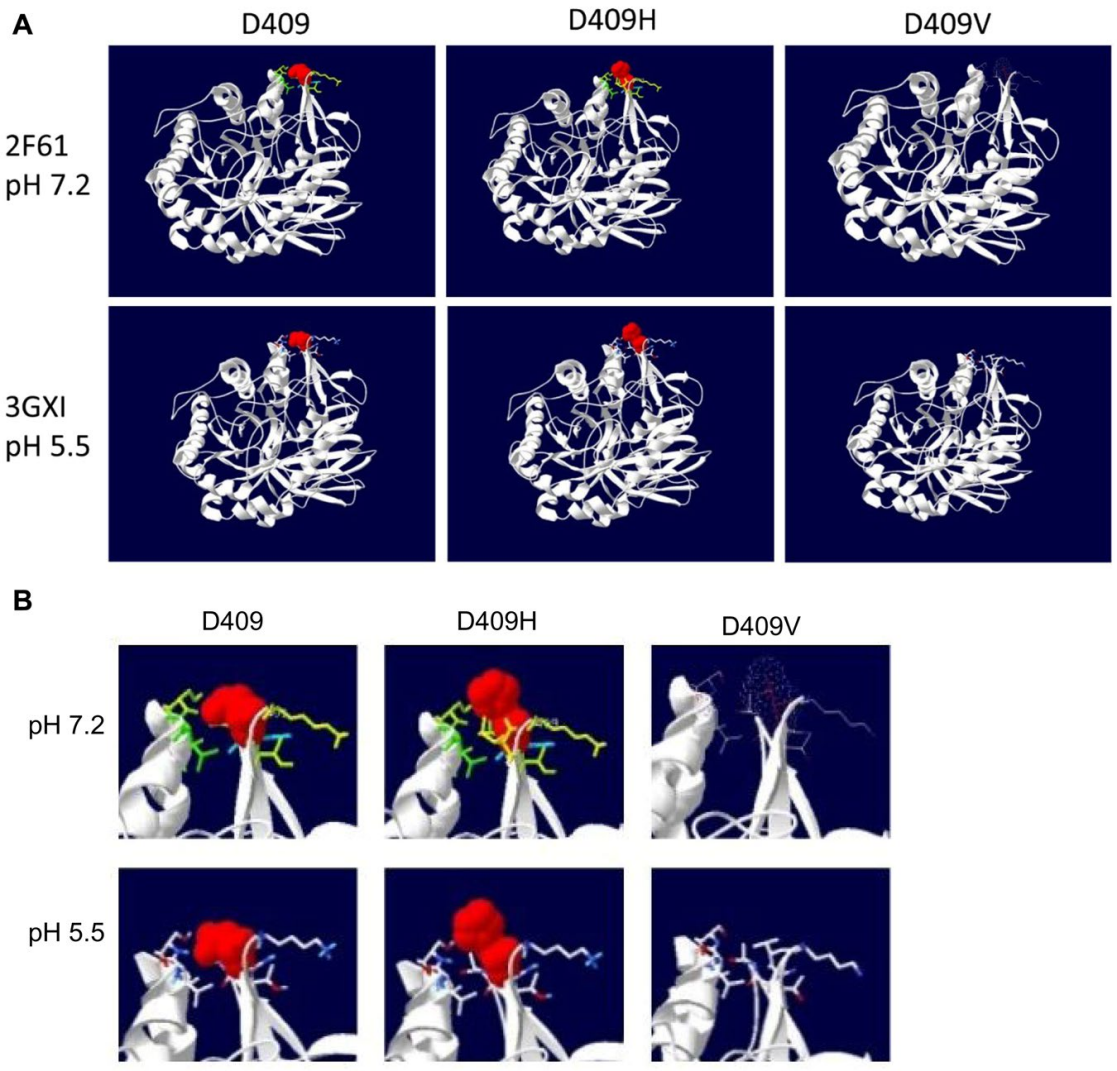
Modeling of mutation effect on GCase structure. (**A**) GCase structure showing side chain interaction with D409. (**B**) Enlarged side chain interaction region. Red clouds show electronic force field at position 409. WT D409 at pH7.2 or pH 5.5 maintain the same side chain interactions with 7 surrounding amino acids showing green/yellow at pH 7.2 and white/blue at pH5.5. Carbons on amino acid are labeled as red dots. D409H gains additional side chain interactions at pH7.2 and turns more dramatic alterations in its conformation at pH 5.5. D409V interact with surrounding amino acids. This mutation at 409 changes D to V (Valine), a non-polar amino acid side chain, which lost all WT interactions with surrounding amino acid side chains. Human PDB crystal structures 2F61, pH7.2, 2.5 Å and 3GXI, pH 5.5, 1.84 Å from Swiss PDB Viewer (DeepView, SPDBV,Version 4.10) program were used for modelling. Amino acids involved side chain interaction are listed in Supplementary Table 1.

D409 (wild type, WT) at pH 7.2 and pH 5.5 maintained the same side chain interactions with 7 surrounding amino acids (Fig. 1, Supplementary Table 1). The force field energy for D409 changed from −17.148 KJ/mole at pH 7.2 to −12.592 KJ/mole at pH 5.5 (Supplementary Table 1). Histidine (H) has a polar amino side chain and can either be protonated or deprotonated. D409H showed a gain of an additional side chain interactions with I406 at pH7.2 and involved 5 additional interacting amino acids at pH 5.5 (Supplementary Table 1). Force field energy analyses for D409H showed conversion to an unfavorable energy environment (positive), 17.879 KJ/mole at pH7.2 and 10.184 KJ/mole at pH 5.5, suggesting this mutation would produce a significant conformational change and altered side chain interactions in neutral and acidic environments. The D409V contains a non-polar amino acid, valine, which lost all WT interactions with surrounding amino acid side chains (Fig. 1, Supplementary Table 1). The force field energy value for D409V was positive, i.e., unfavorable, 56.649 KJ/mole at pH 7.2, and more unfavorable, 72.925 KJ/mole at pH 5.5, relative to WT and D409H. These results indicate that D409V has greater unfavorable energy environment than D409H that could result in their differential *in vivo* hydrolytic properties. D409 is within the GCase binding motif (_399_DSPIIVDITKD_409_) for LIMP-2 (lysosomal integral membrane protein 2), the internal trafficking chaperone of GCase for lysosomal targeting^28,29^. Mutation at this position (D409V, D409H) leads to an unstable enzymes that predisposed them to protease degradation compared to WT.

V394 is located on Domain 1 of GCase at the anti-parallel β-sheet, which is close to the active site pocket (a loop formation) opening^13,30^. The amino acids involved in the side chain interaction in V394L mutant are different from WT (Supplementary Table 1). The force field energy calculated at the WT, i.e., V394, is dramatically increased from 11.884 to 2627.135 KJ/mole (220 fold) for V394L mutant at pH 7.2 (Supplementary Table 1). At pH 5.5, the force field energy for V394L mutant changes from 12.841 to 28645.000 KJ/mole (2230 fold) compared to WT, especially in the non-bound energy calculation, suggesting this mutation affects GCase both in structure as well as accessibility for side chain interaction leading to reduced enzymatic activity.

The side chain interaction at N370S was analyzed based on its crystal structures at pH 7.1 (3KEH) and acidic pH 5.4 (3KEO)^18^. Located at Domain III, the role of N370 in the catalytic cycle is significant, probably associated with local conformational effects at or near the active site^15^. Here, N370S at pH 5.4 showed slight conformation changes compared to WT (Supplementary Table 1). The WT N370 has a negative energy force (−193 to −199 KJ/mole), which may allow the water solvent to maximize its entropy, lowering the total free energy at this region toward catalytic function. Once it was mutated into N370S, the energy force shifts to higher entropic stage (−16 and −26 KJ/mole) and sequentially alters the local conformational (side chains) interaction that could contribute to reduced catalytic activity.

The simulation analyses suggest that each mutant leads to different alteration on side chain interaction, which may underlie the phenotypic variation.

### Mouse models of homozygotes for *Gba1* mutations

The mouse models having the GCase mutations, 9H, 9V, 0S and 4L, were generated previously to study their *in vivo* effects^12^. Homozygotes for 9V, 9H and 4L in mice had reduced tissue GCase activity (Table 1). However, these mutant mice at about 1 year of age do not accumulate significant levels of substrates, do not develop severe CNS and visceral phenotypes, and have normal life spans (Table 1)^12,16^. Mice homozygous for 0S die within the first 24–48 hrs of birth, due to skin permeability defects^12^. Thus, these mutants have limitations in studying the mutations’ *in vivo* effects.

**Table 1:** *Gba1* mutants and Saposin C deficiency models.

Mouse models	Life span	Neuronal phenotype	Storage cells	GCase activity	Glucosylceramide Increase
*Combined mutants*				**vs**. ***Gba1*** **mutant**	**(Brain, Viscera)**
9V;C*	<1 day	un	un	decreased	Yes, Yes
0S;C*	<1 day	un	un	decreased	un, Yes
4L;C*	7 weeks	Yes	un	decreased	Yes, Yes
9H;C*	~13 months	Yes	Yes	same low level	Yes, Yes
*Gba1 and Saposin C mutants* ^*#*^				**%WT**	**(Brain, Viscera)**
9V/9V (9V)	~24 months	~2 years	un	~5	un, Yes
0S/0S (0S)	<1 day	un	un	~10	un, Yes
4L/4L (4L)	~24 months	un	un	~10	Yes, Yes
9H/9H (9H)	~24 months	un	un	~5	un, Yes
WT;C*	~24 months	Yes	un	~40	un, un

un, undetectable.

^#^Some of data are from previous publications^12,16,26,27^.

### Double homozygotes for *Gba1* mutations and Saposin C deficiency

**Figure 2:**
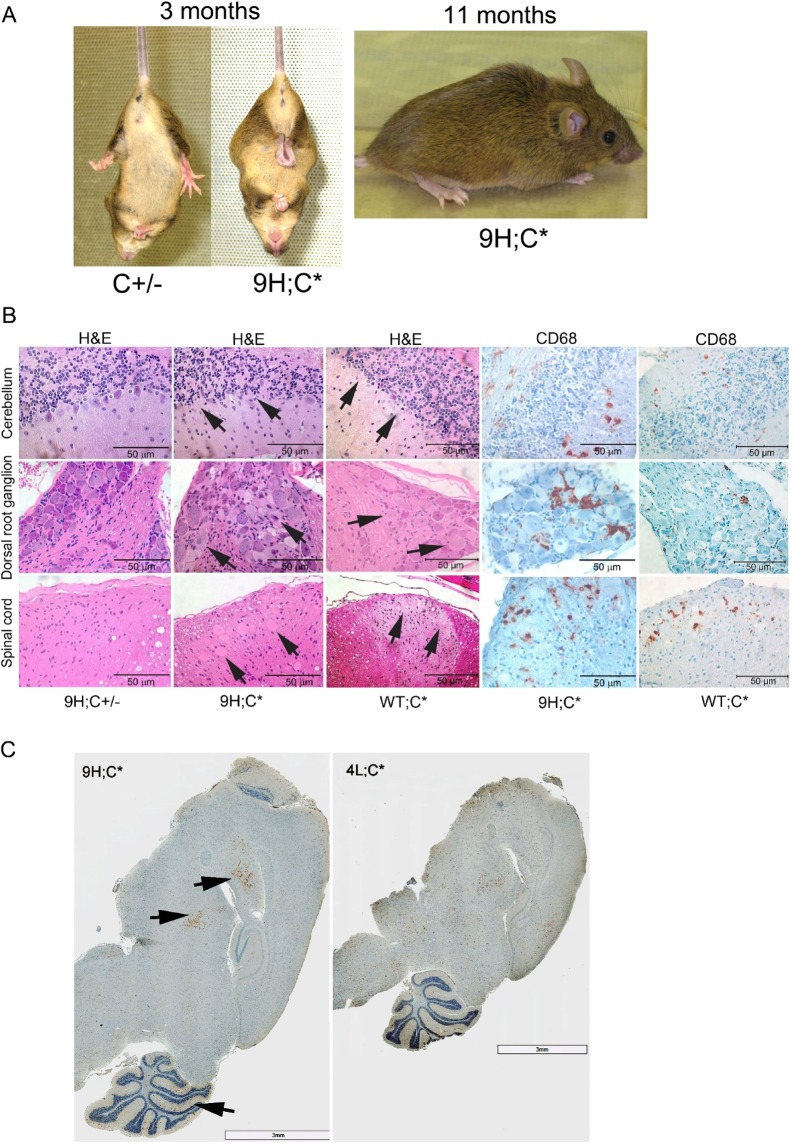
CNS pathology in 9H;C* mice. (**A**) Phenotype. 9H;C* mice showed hind-limb clasping during tail hanging at 3 months of age (left panel) and kyphotic posturing at 11 months of age (Right panel). As a control, C+/− mouse did not show hind-limb clasping. (**B**) CNS pathology in 9H;C* mice compared to WT;C* and 9H;C+/− control mice at 12 months of age. (Top panels) Loss of Purkinje cells (H&E, arrows) was evident in 9H;C* and WT;C* cerebellum and was accompanied with activated microglial cells positive for anti CD68 antibody (CD68, brown) staining. (Middle panels) Dorsal root ganglion in 9H;C* mice contained foamy storage materials in cells (H&E, arrows) and had CD68 positive cells (brown). (Lower panels) Dorsal horn of spinal cord in 9H;C* mice had axonal spheroids (H&E, arrows) and CD68 positive cells (brown). WT;C* mice had fewer foamy cells, axonal spheroids and CD68 positive cells than 9H;C* mice. As a control, 9H;C+/− mice tissues showed normal histology. (**C**) CD68 positive signals (brown) distributed differently in 9H;C* and 4L;C* brains. CD68 signals were restricted in caudate putamen (cp), thalamus (th) and cerebellum (cb) regions (arrows) in 9H;C* brain and distributed in most regions in 4L;C* brain.

To understand differential *in vivo* effect of these mutations, mice were created with homozygosity for *Gba1* mutations in combination with Saposin C deficiency. Saposin C has activity optimization and protective functions on GCase^23^. Deficiency of Saposin C leads to reduced GCase activity that could potentiate the disease phenotype in *Gba1* mutant mice^26,27^. Four combined *Gba1* homozygous mutation mice with Saposin C deficiency (*Gba1* mutation;C*) were generated (Table 1). The 9V;C* and 0S;C* mice died within 24 hours after birth due primarily to skin permeability abnormalities. 4L;C* mice showed primary CNS deficits and had short life span (~7 weeks); phenotypic and pathologic findings have been published and summarized in Table 1 ^27,31^. The 9H;C* mice developed a neurological phenotype resembled that of WT;C* mice^26^, but with earlier (~3 months) vs. ~8 months (WT;C*) onset and with shorter lifespan (Table 1)^26^. The 9H;C* behavioral phenotype included hind-limb clasping during tail hanging by 3 months and the development of kyphotic posturing at 12 months of age (Fig. 2A). The 9H;C* mice also showed mild hind limb paresis and gait ataxia. Compared to the 7 weeks survival of 4L;C* mice, 9H;C* mice survived to about 13 months of age^27^.

### CNS and visceral histopathology of 9H;C* and 4L;C* mice

9H;C* mice developed severe brain and spinal cord histopathology, similar to, but earlier than, the WT;C* mice, major losses of Purkinje cells in cerebellum, inclusions in dorsal root ganglia, and axonal degeneration in spinal cord by 6 months of age (Fig. 2B)^26^. However, these were about 6 months earlier than the appearance of the corresponding lesions in WT;C* mice^26^. Proinflammatory reactions in 9H;C* mice were shown by positive CD68 staining of activated macrophages (Fig. 2B and C). In comparison to 4L;C* at 45 days of age in which the CD68 signals were distributed throughout the entire brain, the CD68 signals in 9H;C* brains were restricted in thalamus, basal ganglia and dentate nucleus of cerebellum in 9H;C* brain at 1 year of age (Fig. 2C). Proinflammation was observed in spinal cord in both 9H;C* and 4L;C* models (Fig. 2B)^27^.

**Figure 3:**
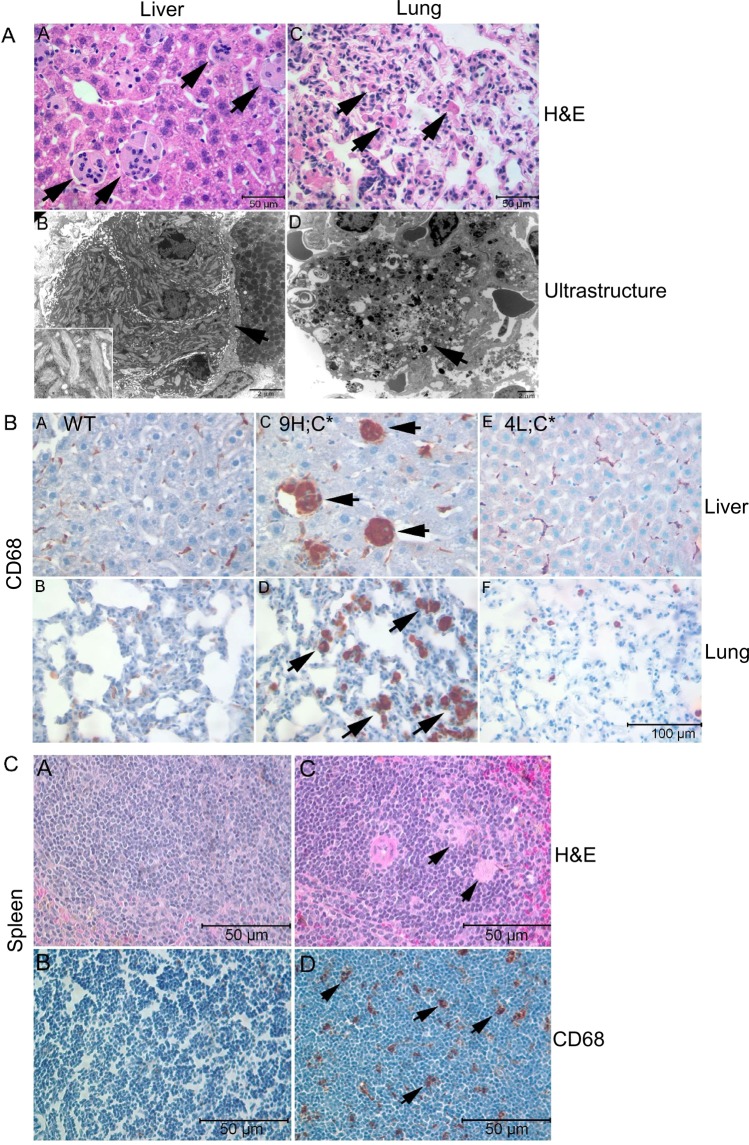
Visceral pathology of 9H;C* mice. (**A**) H&E stained 9H;C* liver and lung at 13 months of age showed storage cells (arrows) in the liver (A) and lung (C). Ultrastructural studies demonstrated the storage cells form multi nucleic cluster in the liver (B). The storage materials had tubule form (B insert). The membrane inclusions were in the lung storage cells (D). (**B**) Anti-CD68 antibody (brown) stained liver and lung. 12-month WT mouse liver (A) and lung (B) showed background level of CD68 signals. 9H;C* liver (C) and lung (D) at 12 months of age had engorged CD68 positive macrophages (arrows). 4L;C* liver (E) and lung (F) at 45 days of age did not have storage cells. Scale bar = 100 µm for all images. (**C**) Compared to age-matched WT spleen stained by H&E (A) and anti-CD68 (B), 9H;C* spleen at 12 months of age had storage cells (arrows) by H&E (C) and CD68 positive cells (D).

Visceral involvement was also present in the 9H;C* mice. Engorged macrophages were observed in liver, lung and spleen (Fig. 3). In contrast to 4L;C* livers and lungs that had WT level CD68 signals and no storage cells (Fig. 3B), massive CD68 positive storage cells were in 9H;C* liver, lung and spleen (Fig. 3B and C). Electron micrographs of storage cells showed typical tubular structure to the accumulated materials which resembled of the storage materials in human GD Kupffer cells and other macrophages (Fig. 3A-B)^4^. In lung, there were many dense aggregates and membrane like materials in interstitial or alveolar macrophages (Fig. 3A–D).

### Skin histopathology of 9V;C* and 0S;C* mice

**Figure 4:**
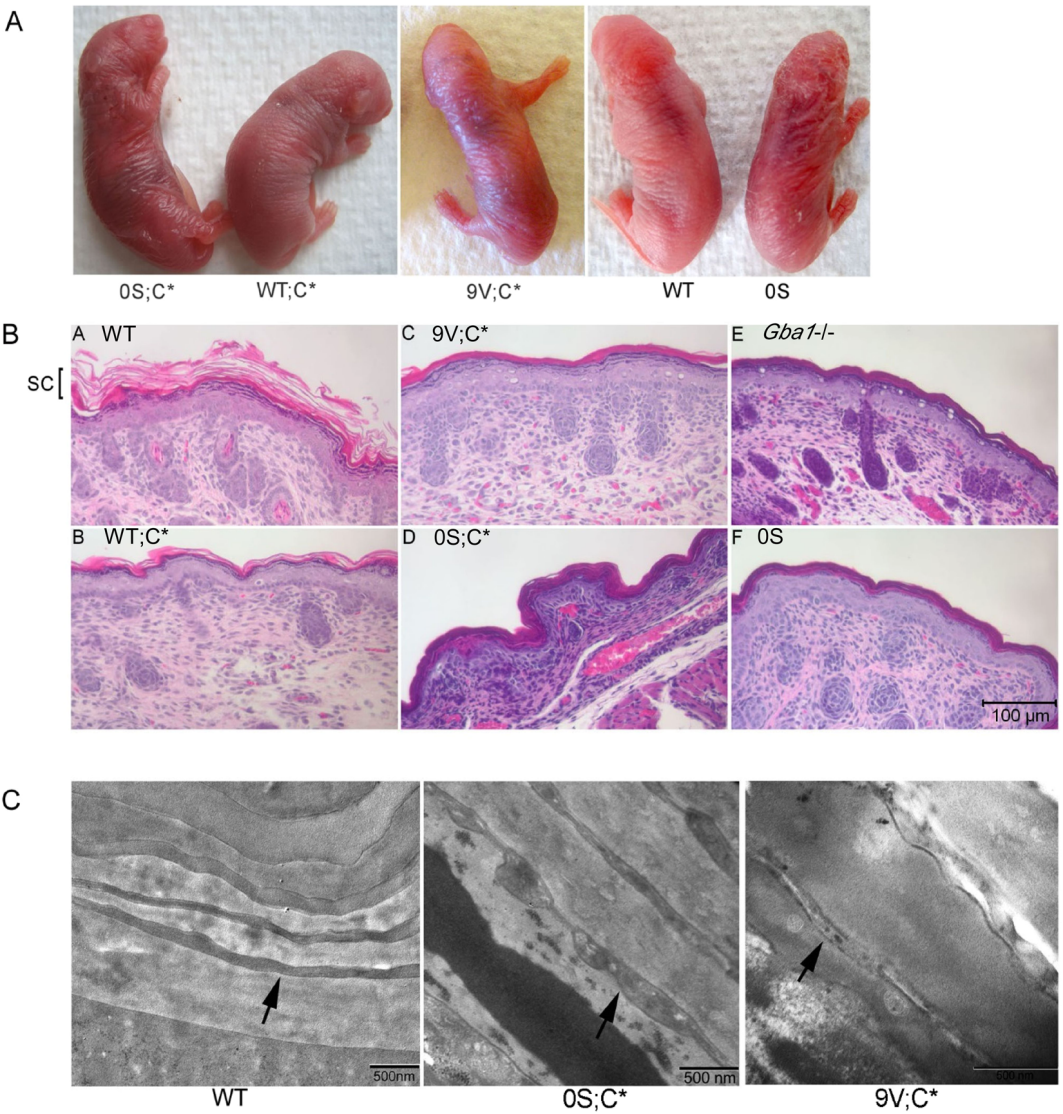
Skin of 9V;C* and 0S;C* mice. (**A**) 9V;C*, 0S;C* and 0S pups had ichthyotic skin compared to smooth skin of WT and WT;C* pups. (**B**) H&E stained skin epidermal sections from 1-day old pups. Normal stratum corneum (SC) in WT (A) and WT;C* (B) skin had basket weave appearance. SC was compact in 9V;C* (C), 0S;C* (D), *Gba1*−/− (E) and 0S (F) skin epidermis. (**C**) Ultrastructural studies of stratum corneum in 1-day old pup skin. Normal lamellar structure (arrow) in WT skin. 9V;C* and 0S;C* had loosely packed layers and irregular lamella structure (arrow).

Similar to 0S and *Gba1*−/− (i.e., GCase null) pups, 9V;C* and 0S;C* mice died within 24 hours of birth^12^. 9V;C* and 0S;C* pups had ichthyotic skin with wrinkly appearance, compared to smooth skin in WT and WT;C* pups at 1 day of age (Fig. 4A). By H&E staining, skin from WT and WT;C* pups had normal stratum corneum showing a basket weave appearance (Fig. 4B), whereas this layer was compact in 9V;C* and 0S;C* skin, which was very similar to that in *Gba1*−/− and 0S skin epidermis (Fig. 4B). Ultrastructural analyses of stratum corneum from 9V;C* and 0S;C* mice showed loosely packed layers and irregular lamella structure (Fig. 4C). In comparison, the corresponding WT pup skin had a lamellar structure. Histology of 9V;C* and 0S;C* visceral organs and brain appeared normal, e.g., no storage cells were found in liver, spleen and lung of 9V;C* and 0S;C* pups.

### GCase activity deficiency in combined *Gba1* mutation and Saposin C deficient mice

**Figure 5:**
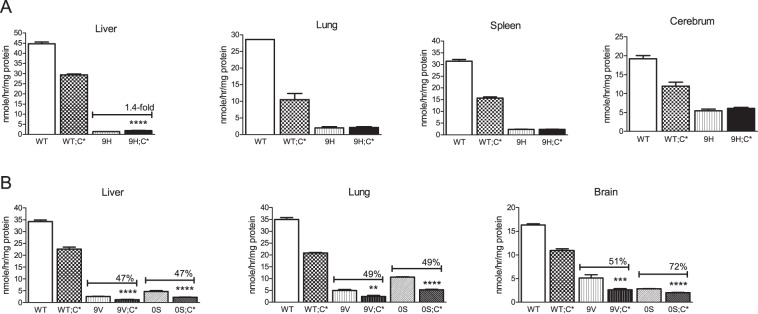
GCase activity. (**A**) GCase activity in 9H;C* mice tissues were reduced compared to WT and WT;C* mice. Compared to 9H mice, GCase activity in 9H;C* tissues were not changed in the lung, spleen and cerebrum, but slightly elevated in the liver. (**B**) 9V;C* and 0S;C* mice had reduced GCase activity in the liver, lung and brain compared to 9V and 0S tissues, respectively. Student’s t-test (n = 3–6 mice).

GCase activity in the tissues of the *Gba1* mutation;C* mice were compared to those from WT and WT;C* mice. Consistent with previous studies, GCase activity was reduced by ~40% in the organs of WT;C* mice (Fig. 5)^26^. In 9H;C* mice, GCase activity in liver, lung, spleen and cerebrum were about or less than 5% of WT level, but at similar levels as that in 9H tissues, although at these very low activity levels are difficult to compare directly (Fig. 5A). 9H GCase is a very unstable protein^13^. The similarity of GCase activity in 9H;C* and 9H tissues suggest that may not reflect potential differences detectible *in vivo*, i.e., the very low *in vivo* levels are not reflected by the *in vitro* activity assessments. Compared to 9V mice, 9V;C* had apparently reduced GCase activity in liver, lung and brain (Fig. 5B). 0S;C* mice also showed similarly decreased activity in liver, lung and brain compared to 0S/0S tissues (Fig. 5B). Reduced GCase activity by ~50% was reported previously in 4L;C* compared to 4L tissues^27^. These results showed that Saposin C deficiency in 9V, 0S and 4L mice leads to reduction of mutant GCase activity.

### Analyses of substrate levels in the mice tissues

**Figure 6:**
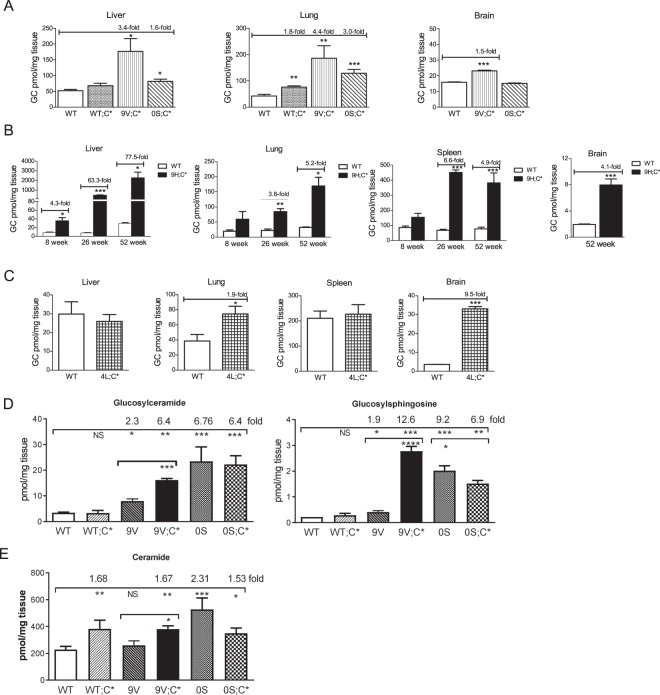
Tissue GC and GS analysis by LC/MS. (**A**) 9V;C* and 0S;C* mice had GC accumulation in liver and lung. 9V;C* had GC accumulation and 0S;C* mice had WT GC level in brain. (**B**) 9H;C* visceral and brain tissues showed GC accumulation increased with age. (**C**) GC levels were increased in 4L;C* brain and lung compared to WT mice. **(D)** Epidermal GC and GS levels were significantly increased in 9V;C*, 9V, 0S;C* and 0S mice at 1 day of age compared to WT. Epidermal GC and GS levels in 9V;C* were higher than 9V. (**E**) Total ceramides were significantly increased in 0S, 0S;C*, 9V;C* and WT;C* mice epidermis compared to WT. Epidermal ceramide levels in 9Vmice were not significantly different from WT. Student’s t-test (n = 3–6 mice).

GC levels in 9V;C* and 0S;C* tissues were compared to WT and WT;C* mice at 1 day of age. 9V;C* and 0S;C* liver and lung had significantly increased GC compared to WT (Fig. 6). 9V;C* brain showed a 1.5-fold increase in GC above WT level (Fig. 6A). GC levels in 0S;C* brain were comparable to the WT level (Fig. 6A). GS levels in 9V;C* liver, lung and brain and 0S;C* lung were detectable and slightly above WT level (Table 2).

**Table 2:** Glucosylsphingosine levels (pmol/mg tissues).

Mouse models	Age	Liver	Lung	Spleen	Brain
WT	<1 day	un	un	ND	un
WT	6 weeks	un	un	un	1.7 ± 0.1
WT	52 weeks	un	un	un	2.7 ± 0.3
9V;C*	<1 day	1.2 ± 0.1	1.9 ± 0.7	ND	0.7 + 0.0
0S;C*	<1 day	un	0.9 ± 0.1	ND	un
4L;C*	6 weeks	1.5 ± 0.4	1.9 ± 0.9	2.4 ± 0.2	12.3 ± 0.2^^^
9H;C*	52 weeks	un	un	1.6 ± 0.7	5.4 ± 0.3^^^

un, undetectable.

^^^significant difference compared to age-matched WT.

ND, not determined.

In 9H;C* mice GC levels were massively increased in liver and moderately elevated in lung, spleen and brain (Fig. 6B). The GC levels in 9H;C* visceral tissues increased with age (Fig. 6B). In contrast to 4L;C* mice that had higher GC accumulation in brain than viscera (Fig. 6C and Table 1)^27^, 9H;C* had a greater GC content in visceral tissues than brain. GS levels in 9H;C* mice were also higher in both visceral tissues and brain (Table 2).

GC and GS in skin epidermis of 1-day old 9V;C* and 0S;C* pups were compared to age-matched WT, WT;C*, 9V and 0S mice. 9V;C* epidermal GC and GS were significantly increased compared to WT, WT;C* or 9V (Fig. 6D). GC and GS accumulated in 0S;C* epidermis and levels were significantly higher compared with WT and WT;C* mice (Fig. 6D). GC and GS levels were comparable in 0S;C* and 0S epidermis, but higher in 9V;C* than 9V (Fig. 6D). The accumulated epidermal GC species detected ranged from GC16:0 to GC30:0 with the major accumulated species being GC16:0 and GC26:0 (Supplementary Fig. 1A). Ceramide levels in 0S epidermis were 2-fold above WT levels, however, ceramide levels in 9V;C*, 0S;C* and WT;C* were increased less than 2-fold from WT level, but not increased in 9V epidermis (Fig. 6E). Ceramide species profile revealed that C16-OH, C24 and C26-1 are the major species in mouse epidermis. Various ceramide species were increased in those mutants compared to WT mice epidermis (Supplementary Fig. 1B).

In summary, simulation analysis suggest the conformational alterations in 9H, 9V, 4L and 0S mutant GCase results in reduced enzymatic activity and stability. Although the homozygous *Gba1* mutations in mice develop relatively mild phenotypes, deficiency of Saposin C in *Gba1* mutants potentiate the disease and reveal mutation-dependent phenotype variation.

## Discussion

The findings from our studies suggest GCase structure altered by specific mutations exhibits differential phenotypes in Gaucher disease. Enzymatic properties of the mutant 9H, 9V, 4L and 0S have been characterized in human recombinant proteins and mouse fibroblasts^13^. Using non-natural substrates *in vitro*, 9H and 9V GCases have ~5% of WT GCase activity and are unstable within cells, whereas 0S and 4L mutants are stable, but have reduced catalytic activity to ~12–14% of WT levels. 0S and 4L mutations have similar enhanced activation by the phosphatidylserine (PS) induced conformational change, whereas Saposin C’s activation effects on these GCases was similar to WT levels^13^. The *in vivo* outcome of these mutants were explored in the mice with homozygosity for a specific *Gba1* mutation together with Saposin C deficiency. Compared to point mutated *Gba1* mutant mice, 9H, 9V, 0S and 4L, backcrossing of C* mice with those *Gba1* mutants produced the 9H;C*, 4L;C*, 9V;C* and 0S;C* mice with additionally reduced mutant enzyme activity and increased substrates levels. Saposin C deficiency did not change the phenotype in 0S due to early death from the skin permeability abnormality in 0S mice^12^. In 9H;C*, 9V;C* and 4L;C* mice, the deficiency of Saposin C potentiated the disease progression in the variant phenotypes.

The distinct phenotypes from 9V;C* (1-day survival) and 9H;C* (13 months survival) mice suggest that D409 is a critical amino acid affecting catalytic function of GCase. Aspartic acid (D) has an acidic/negatively charged side chain, whereas histidine (H) has a basic/positively charge side chain, and valine (V) is a non-polar amino acid. GCase has three domains resolved by X-ray crystallization. Domain 1 (residues1–27 and 383–414), where D409 resides, is predicted to be important in enzyme folding and stability, domain 2 (residues 30–75 and 431–497) has an immunoglobulin-like structure, and domain 3 (residues 76–381 and 416–430) forms TIM barrel-helix6 and helix7 consisting of catalytic site^13,30^. LIMP2 is an intracellular receptor for lysosomal targeting of GCase^28,32^. The binding motif of GCase for LIMP2 is contained in the span covered by amino acids 399 to 409 in Domain III of GCase^28,29^. The mutant GCases, D409V or H, disrupts lysosomal targeting of these GCases leading to their proteolysis. The crystal structure of mouse GCase is presently not available, but is predicted to be very similar to the human WT enzyme. Human and mouse GCases are highly homologous, have 85% amino acid identity, normal and mutant GCases, e.g., 4L and 9V and 9H, have similar respective properties^13^. These similarities provide the basis for modeling of the human GCase structures at neutral and acidic pH to simulate mutation effects *in vitro* (human) and *in vivo* (mouse) of a given amino acid and to gain insight into GCase’s function. Analysis of side chain interaction and its force field energy indicates that D409 has side chain interaction with N19 (within 6 Å), a critical glycosylation site in GCase function^13,33^. The 9V mutation abolished nearly all side chain interactions in Domain 1 and the favorable energy environment, which leads to greater conformational changes at pH 5.5. In comparison, 9H has a significant conformational change and altered side chain interaction in an acidic environment. 9V and 9H mice have similar mild phenotypes up to 12 months^12^, but the interaction with Saposin C deficiency brings out the differential properties of the GCase mutant enzyme alone without the protective effects of Saposin C. This simulation result supports the differential phenotype of two mutants with severe, early death of 9V;C* and mild, chronic form of 9H;C*.

The V394 is located in Domain 1 close to the active site pocket. The modeling of V394L implicates an altered conformation change of this GCase leading to its functional effects on catalysis. The force field energy at V394 is dramatically increased when Valine was replaced by Leucine (V394L) at pH 7.2 and pH 5.5, indicating significant alterations of the side chain geometries with resultant negative effects on active site function. N370 is located in Domain 3 on the helical turn near loop1 and contributes to the hydrogen bonding network to stabilize the orientation of this loop. The residue Y313 in loop 1 (residues 311–319) plays a key role in moving this loop to open the active site for binding^15^. At pH 7.2, the N370 side chain forms a stable interaction with L310 (Table 1) and at pH 5.5, N370 forms an even more stable interaction with W312 and D315 (Table 1, red) to stabilize the Y313 (a chelated-like interaction). Mutation of 370 from N to S alters the original with L310 to W312 and forms two new interactions with V376 and G377. This is a significant alteration of loop 1 (amino residues 311–319) structure. The serine side chain of N370S at acidic pH 5.4 cannot form a chelated-like interaction with W312 and D315 and sways the contact with W312 and A320. This has been suggested to affect substrate access to the active site^15,34^. The simulation analyses of V394L and N370S support the structural alteration change on mutated GCase induced enzymatic function deficit. The abnormal skin permeability in 0S and 0S;C* mice prevented the investigation of disease development on this mutant. Whereas 4L mice have nearly normal phenotype and life span^12^, the effect of 4L on disease phenotype was revealed in 4L;C* mice in the absence of Saposin C.

The mouse models developed here not only underscore the *Gba1* mutation on disease development but also provide insight into the *in vivo* role of Saposin C for mutant GCase function. Saposin C exists as a dimer and is required by GCase for optimal activity and protein stabilization against proteolysis^21,23^. Mutated Saposin Cs, which have been cleaved from prosaposin, i.e., the mature form, are unstable and rapidly degraded^35^. Mutations in Saposin C lead to a rare form of Gaucher-like disease presenting neuronopathic and non-neuronopathic symptoms^24,36–38^. The mouse models of Saposin C deficiency develop slow progression of neurological phenotypes, but no substrate and storage cells in visceral organs^26,39^. In Saposin C deficient mice, ~40% reductions of WT or mutant GCase proteins and activities are due to increased GCase proteolysis in the lysosome^23,26,27^, i.e., the loss of Saposin C’s protective effects. The exact mechanism and mode of interaction of GCase and Saposin C has not been fully defined. Saposin C has membrane lipid binding properties and forms a protein complex with GCase at the lipid bilayers, as demonstrated by *in vitro* experiments^40,41^. Saposin C plays role in lipid presentation by CD1b, the molecule responsible for lipid-antigen presentation to T-cells in immune response^42,43^. Saposin C’s membrane interactions are required for providing GCase accessibility to its substrates, GC and GS embedded in the membrane^44,45^. Studies of V394L with PS or Saposin C reveal a surface-accessible loop structure containing V394 (394–414) sensitive to PS and Saposin C activation^13^. Together with D409 in the same loop, this region exhibits sensitivity to conformational changes altering protein stability and activity as well as activator interactions (e.g. PS and Saposin C)^28^. How Saposin C deficiency differentially affects 9H or 9V is not known, partially due to their instability during purification, which inhibits direct modeling studies. However, 3D-Docking models of GCase and Saposin C predicts interactions with domain 2 and helix 6 of domain 3 on GCase^46^. Both D409 and V394 are not within those domains. Recent structural study showed Saposin A binds its cognate enzyme galactocylceramidase and form a heterotetramer complex^47^. Further studies of the structural complex of Saposin C and GCase will be needed to resolve the mechanism and mode of interaction.

Anionic phospholipids-containing membranes are essential for Saposin C’s function^40^. Phospholipid composition could influence Saposins C’s action and affect its interaction with GCase, consequently mutated GCase may have defect in response PS or Saposin C’s activation^13^. By reducing anionic phospholipids to 20% of total lipids, Saposin C promotes binding and activation of normal GCase, but loses its effects on N370S GCase^48^. The different phenotype of 9H;C*, 4L;C* or 9V;C* mice could be influenced by phospholipids compositions that affect the interaction of Saposin C and mutant GCase in specific cells/organs.

The different phenotyps of these mutant mice could be explained by changes in substrate specificity of mutant GCase. GCase has two substrates: GC and GS. GCs are a group of lipids containing glucose and ceramide with fatty acids of various chain length^16,49^. GS is deacyl form of glucosylceramide resulting from acid ceramidase hydrolysis^50,51^. Degradation of GC requires three components, GCase, Saposin C and phospholipids^52,53^. It is evident that there is mutation-specific quantitative differences in GC species and GS accumulations that influences tissue/regional expression of Gaucher disease phenotypes in these mouse models^16^. 4L;C* mice had major GC18:0 degradation defects in the brain, whereas the analogous mice with 9H;C* led to all GC species accumulating massively in visceral tissues^16^. GS was poorly degraded in brain by 4L and 9H GCases, but not by 9V and 0S GCase. Such differences are anticipated from the basic kinetic properties of the variant GCases, and the ratio of the rate constants for the cleavage of GC and GS^54^. Saposin C could also influence the substrate preferences for various GCase variants *in vivo*.

Defective GCase activity in the hydrolysis of GC to ceramide may affect maturation or change in lipid membranes that form the normal epidermal barrier^55^. In both Gaucher disease type 2 patients and *Gba1* knock out mice, epidermal abnormalities are associated with the accumulation of GC^56–58^. The 9V;C* and 0S;C* pups showed compact stratum corneum structure and irregular layers of lamellar body. Significantly increased epidermal GC and GS likely account for the epidermal abnormalities in those mice skins. Unexpectedly, the ceramide level was not significantly reduced in those mice although GC was increased, which is in contrast to previous report of reduced ceramide in Gaucher disease type 2 patient and in knock out mice^56–58^. In those studies, total GC and ceramides were determined by thin layer chromatography^56–58^. In current study, epidermal glycosphingolipids were quantitated by LC/MS. With the available ceramide standards, the detectable longest fatty acid chain length ceramide was C18:1/24:0, which may omit long chain and complex ceramide that contribute to major portion of ceramides in epidermis^59^. With species < GC24, accumulated GCs did not lead to reduction of same chain length of ceramide. In addition, this study demonstrated that deficiency of Saposins C alone did not cause GC and GS accumulation and abnormal stratum corneum in epidermis which is dissimilar to deficiency of prosaposin that leads to aberrant lamellar membrane structure and GC accumulation^60^.

In summary, 9H;C*, 9V;C*, 0S;C* and 4L;C* mice provide additional *in vivo* models to study the tissue specific pathogenic effect of *GBA1* mutations, e.g. 9V;C* and 0S;C* for skin permeability, 4L;C* for neuronopathic phenotype^27^ and 9H;C* for chronic form of both viscera and brain pathology in Gaucher disease. Valvular disease found in human patients^10^ was not observed grossly in 9H^12^ or 9H/C* mice which presents a limitation for studying valvular disease using these models. Biochemical and pathological data from this study support a functional interaction of GCase and Saposin C. Without Saposin C, the *Gba1* mutant mice developed mutation-dependent and tissues-specific phenotypes. The results of these studies provide insights to GCase mutations and their correlation with tissue specific variation in substrate accumulation and disease phenotype, which lays the groundwork for comparative human studies in exploring genotype and phenotype correlations in Gaucher disease.

## Methods

### Mouse models and tissues collection

*Gba1* mutant mice with 9H, 9V, and 0S homozygosity were generated as described^12^. Saposin C deficient mice (WT;C*) were created by knock-in of a point mutation on Saposin C domain of *Psap*^26^. The combined *Gba1* mutation and Saposin C deficiency mice (designated as “Gba1 mutation”;C*) were generated by cross-breeding of C* mice with specific *Gba1* mutant mice^26^. The resultant doubly homozygous mice are designated 9H;C*, 9V;C*, and 0S;C*. Generation of 4L;C* mice was described previously^27^. Saposin C heterozygous mice (C+/−) and 9H mice with C+/− (9H;C+/−) did not show abnormal histology or a behavioral phenotype and were used as controls. The strain backgrounds of the various mutant mice and WT mice were C57BL/129. The mice were maintained in microisolators in accordance with institutional guidelines under IACUC approval at Cincinnati Children’s Hospital Research Foundation. The tissues were collected from adult mice after transcardial perfusion with saline and from pups without perfusion. The collected tissues were stored at −80 °C for enzyme and lipid analyses or fixed in fixative for histology studies.

### Histopathological analyses

Mouse tissues were fixed in 10% formalin and embedded in paraffin. The tissues were sectioned and stained with Hematoxylin and Eosin (H&E) and analyzed under light microscopy. Karnovsky’s fixative was used for ultrastructural studies. For immunohistochemistry, frozen tissue sections fixed with 4% paraformaldehyde were incubated with rat anti-mouse CD68 monoclonal antibody (Serotec, Oxford, UK) at 1/200 dilution in PBS with 5% BSA overnight at 4 °C as described^22^. Detection was performed using ABC Vectastain and Alkaline Phosphatase Kit II (Black) according to the manufacturer’s instruction. The slides were counterstained with Hematoxylin.

### Enzyme activity

Tissues were homogenized in 1% Na taurocholate and 1% Triton X-100, with 0.25% each in final assay mixtures. GCase activities were determined fluorometrically with 4- methylumberlliferyl-β-D-glucopyranoside (4MU-Glucose) (Biosynth AG, Switzerland) in the presence and absence of the GCase irreversible inhibitor, 1 mM Conduritol B epoxide (Millipore. CA)^61^. WT GCase activities in control tissues were run in parallel^62^. Protein concentrations were determined using BCA Protein Assay Reagent (Pierce, Rockford, IL).

### Lipid analyses

Tissue glycosphingolipids were extracted in methanol/chloroform/water (2:1:0.7) as described previously^63^ and subjected to alkaline methanolysis and desalting on Sephadex G-25 fine columns. The extracted samples were taken up in methanol containing an internal standard. GC and GS analyses were carried out by ESI-LC-MS/MS using a Waters Quattro Micro API triple quadrupole mass spectrometer (Milford, MA) interfaced with an Acquity UPLC system^16^. Quantification of GCs with various chain lengths was achieved by interpolation of calibration curve for the natural GCs with the most closely related fatty acid chain length. The quantification of GS was based on the curve using GS-d5 (Avanti) as internal standard. Linear responses for GCs and GS were in the range of 25 pg–10 ng.

Epidermis was collected from newborn pup abdomen skin. The skin samples (~1 cm^2^) were incubated with 1 mL of 0.25% Trypsin at 4 °C for 18 hours. Epidermis was separated from tissues and stored at −80 °C for lipid extraction^59^. Glycosphingolipids in the epidermis were extracted with 2 mL of chloroform/methanol/water (30:60:8), sonicated for 15 min at 50 °C, and centrifuged for 5 min at 1000 × g. The extraction was repeated 3 times. The combined extracts were dried under N_2_ followed by dissolving in 5 mL of chloroform/methanol/water (2:1:0.15) and subjected to alkaline methanolysis as described above. The extracted epidermal glycosphingolipids from 1 mg epidermis were taken up in methanol containing an internal standard for quantitation of GC and GS as above^16^. Ceramides in epidermis were quantitated by LC/MS^64^. Glycosphingolipids levels were normalized by wet tissues weights.

### Simulation analysis of GCase mutation

Human GCase crystal structures, pH7.2 (2F61, 2.5 Å)^13^ and pH 5.5 (3GXI, 1.84 Å)^17^, were used to model the D409 and V394 wild type (WT) GCases and their respective mutant forms, D409H, D409V and V394L. Human GCase crystal structures, pH7.1 (3KEH, 2.5 Å) and pH 5.4 (3KEO, 1.84 Å), were applied for modeling N370 WT and mutant N370S^18^. Swiss PDB Viewer (DeepView,SPDBV,Version 4.10) was applied for structure modeling analysis and GROMOS 96 was used for force field energy computations^65^. The distance parameter for computing interactions of target mutation site was set to 6 Å and force field energy changes within introduced mutation were computed. All amino acids in the side chains that interact with the mutated amino acid at this position (e.g. D409H, V394L and N370S) within 6 Å were listed in Supplementary Table 1. The side chain interactions of mutant were compared to WT GCase structure at each position. Differences on the amino acids involved in the interaction in mutant compared to WT are highlighted in red. The parameters for force field energy computation analysis at given position include the energy in bonds, angles, torsion, improper, non-bonded, electrostatic and constraint energy (K joule/mole or KJ/mole). Negative energy values represent favorable energy environment whereas positive values represent unfavorable energy environment for a given amino acid. The pH environment effects on side chain interactions (acidic, pH 5.5 versus neutral, pH 7.2) were also computed.

### Statistical analysis

The data were analyzed by Student’s t-test or One-way ANOVA test with Dunnett posttest using GraphPad Prism.

## Supplementary information

Supplementary data
