# Supplementary material for: Combination of acid β-glucosidase mutation and Saposin C deficiency in mice reveals Gba1 mutation dependent and tissue-specific disease phenotype
Source: Sci Rep. 2019 Apr 3;9:5571. doi: 10.1038/s41598-019-41914-7 (PMC6447580; doi:10.1038/s41598-019-41914-7)
Supplement: Supplementary file 1 — Supplementary data [file 41598_2019_41914_MOESM1_ESM.pdf]

Combination of acid β-glucosidase mutation and Saposin C deficiency in mice reveals *Gba1* mutation dependent and tissue-specific disease phenotype

Benjamin Liou[1], Wujuan Zhang[2], Venette Fannin[1], Brian Quinn[1], Huimin Ran[1], Kui Xu[1], Kenneth D.R. Setchell[2, 3], David Witte[2, 3] and Gregory A. Grabowski[1,3], Ying Sun[1,3,*]

[1]Division of Human Genetics, Cincinnati Children's Hospital Medical Center, Cincinnati, OH
[2]Department of Pathology and Laboratory Medicine, Cincinnati Children's Hospital Medical Center, Cincinnati, OH
[3]Department of Pediatrics, University of Cincinnati College of Medicine, Cincinnati, OH.

Supplementary Data

Supplementary Fig. 1. **Epidermal GC and ceramide species levels.**

Supplementary Table 1. **Amino acids in the side chains interact with WT or mutated amino acid at 409, 394 or 370 position in GCase**

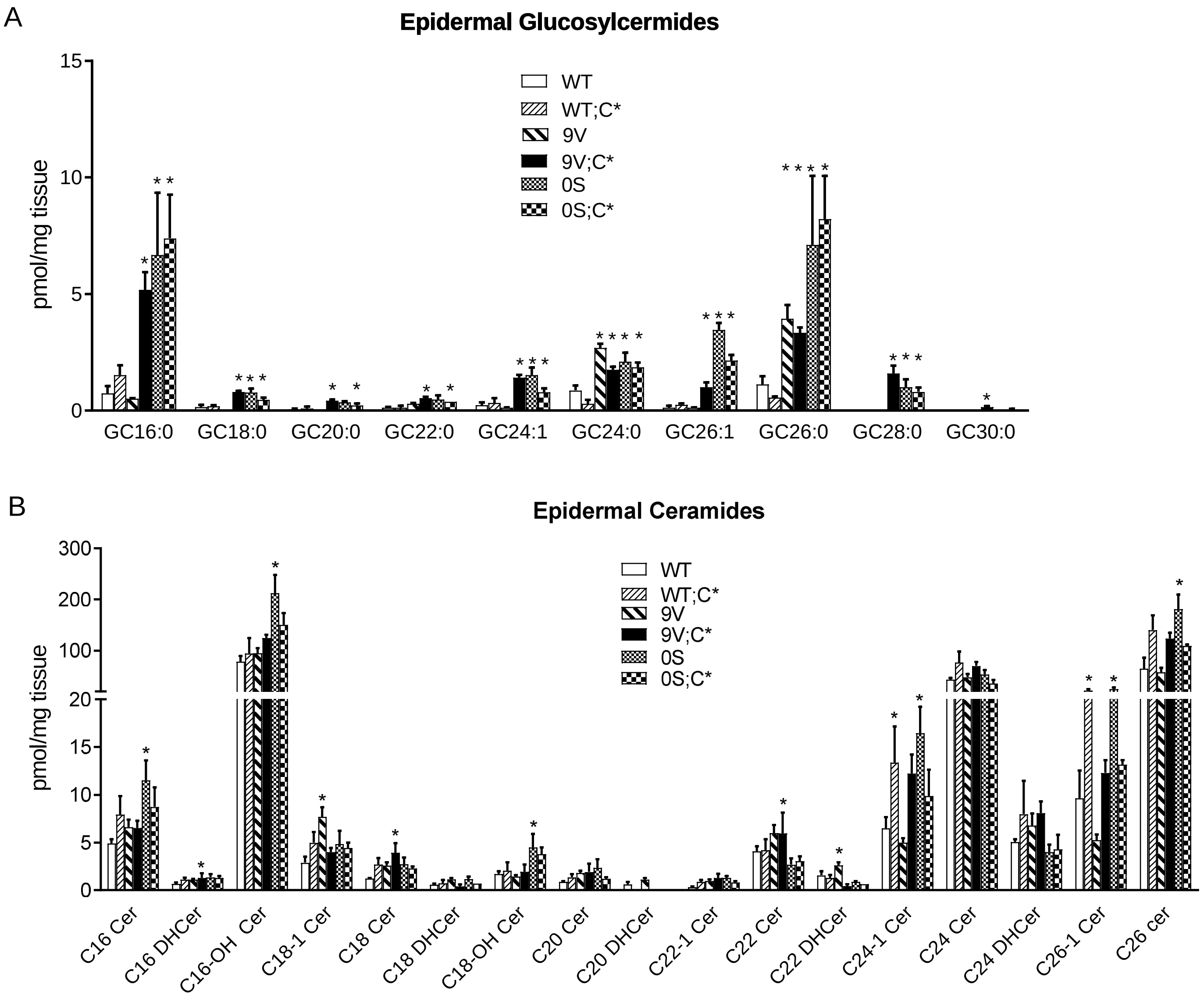

Supplementary Fig. 1. Epidermal GC and ceramide species levels.

(**A**) Various GC species levels were higher in 9V;C*, 9V, 0S;C* and 0S than WT epidermis. (**B**) Ceramides profile shows increased levels of various species in mutant mice compared to WT epidermis. One-way ANOVA with Dunnett posttest. *, $p<0.05$ compared to WT (n=3-5 mice).

Supplementary Table 1. Amino acids in the side chains interact with WT or mutated amino acid at 409, 394 or 370 position in GCase

	**pH 7.2 (2F61, 2.5Å)[1]**	**pH 5.5 (3GXI, 1.84Å)[1]**
Interact with **ASP (D) 409** (WT)[2]	ASN19	ASN19
	ALA20	ALA20
	LEU96	LEU96
	SER97	SER97
	ALA100	ALA100
	LYS408	LYS408
	THR410	THR410
Force field energy (KJ/mole)	**-17.148**	**-12.592**
Interact with **HIS (H) 409** (Mutant)	ASN19	CYS18[2]
	ALA20	ASN19
	LEU96	ALA20
	SER97	ALA95
	ALA100	LEU96
	ILE406	SER97
	LYS408	ALA100
	THR410	VAL404
		ASP405
		ILE406
		LYS408
		THR410
Force field energy (KJ/mole)	**17.879**	**10.184**
Interact with **VAL (V) 409** (mutant)	LYS408	LYS408
	THR410	THR410
Force field energy (Kcal/mole)	**54.649**	**72.925**
Interact with **VAL (V) 394** (WT)	ASP127	ASP127
	PRO245	PRO245
	PHE246	PHE246
	GLN247	GLN247
	ASN392	ASN392
	Ttrp393	Ttrp393
	ARG395	ARG395
Force field energy (KJ/mole)	**11.884**	**12.841**
Interact with **LEU (L) 394** (mutant)	ASP127	ASP127
	ASN129	ASN129
	PRO245	PRO245
	PHE246	PHE246
	GLN247	GLN247
	ASN392	ASN392
	Ttrp393	Ttrp393
	ARG395	ARG395
Force field energy (KJ/mole)	**2627.135**	**28645**

	**pH 7.2 (2F61, 2.5Å)[1]**	**pH 5.5 (3GXI, 1.84Å)[1]**
Interact with **ASN (N) 370** (WT)	GLN76	GLN76
	ARG285	ARG285
	LEU310	TRP312
	LEU324	ASP315
	ALA338	LEU324
	HIS365	ALA338
	SER366	HIS365
	ILE367	SER366
	ILE368	ILE367
	THR369	ILE368
	LEU371	THR369
	LEU372	LEU371
	TYR373	LEU372
	HIS374	TYR373
	VAL375	HIS374
	VAL376	VAL375
	GLY377	VAL376
	TRP378	GLY377
		TRP378
Force field energy (KJ/mole)	**-193.431**	**-199.369**
Interact with **SER (S) 370** (mutant)	**pH 7.1 (3KEH, 2.5Å)[1]**	**pH 5.4 (3KEO, 1.84Å)[1]**
	GLN76	GLN76
	ARG285	ARG285
	TRP312	TRP312
	LEU324	ALA320
	ALA338	LEU324
	HIS365	ALA338
	SER366	HIS365
	ILE367	SER366
	ILE368	ILE367
	THR369	ILE368
	LEU371	THR369
	LEU372	LEU371
	TYR373	LEU372
	HIS374	TYR373
	VAL375	HIS374
	VAL376	VAL375
	GLY377	TRP378
	TRP378	
Force field energy (KJ/mole)	**-16.563**	**-26.06**

1. X-ray structures are referenced Swiss PDB Viewer program.

2. Interaction and force field energy are analyzed by GROMOS 96 algorithm.
3. Amino acids in red are not interact with D409, V394 and N370 on WT GCase.
